# Supplementary material for: Activin-mediated alterations of the fibroblast transcriptome and matrisome control the biomechanical properties of skin wounds
Source: Nat Commun. 2020 May 25;11:2604. doi: 10.1038/s41467-020-16409-z (PMC7248062; doi:10.1038/s41467-020-16409-z)
Supplement: Supplementary file 1 — Supplementary information [file 41467_2020_16409_MOESM1_ESM.pdf]

Supplementary Information

Activin-mediated alterations of the fibroblast transcriptome and matrisome control the biomechanical properties of skin wounds

Wietecha et al.

Supplementary Figure 1

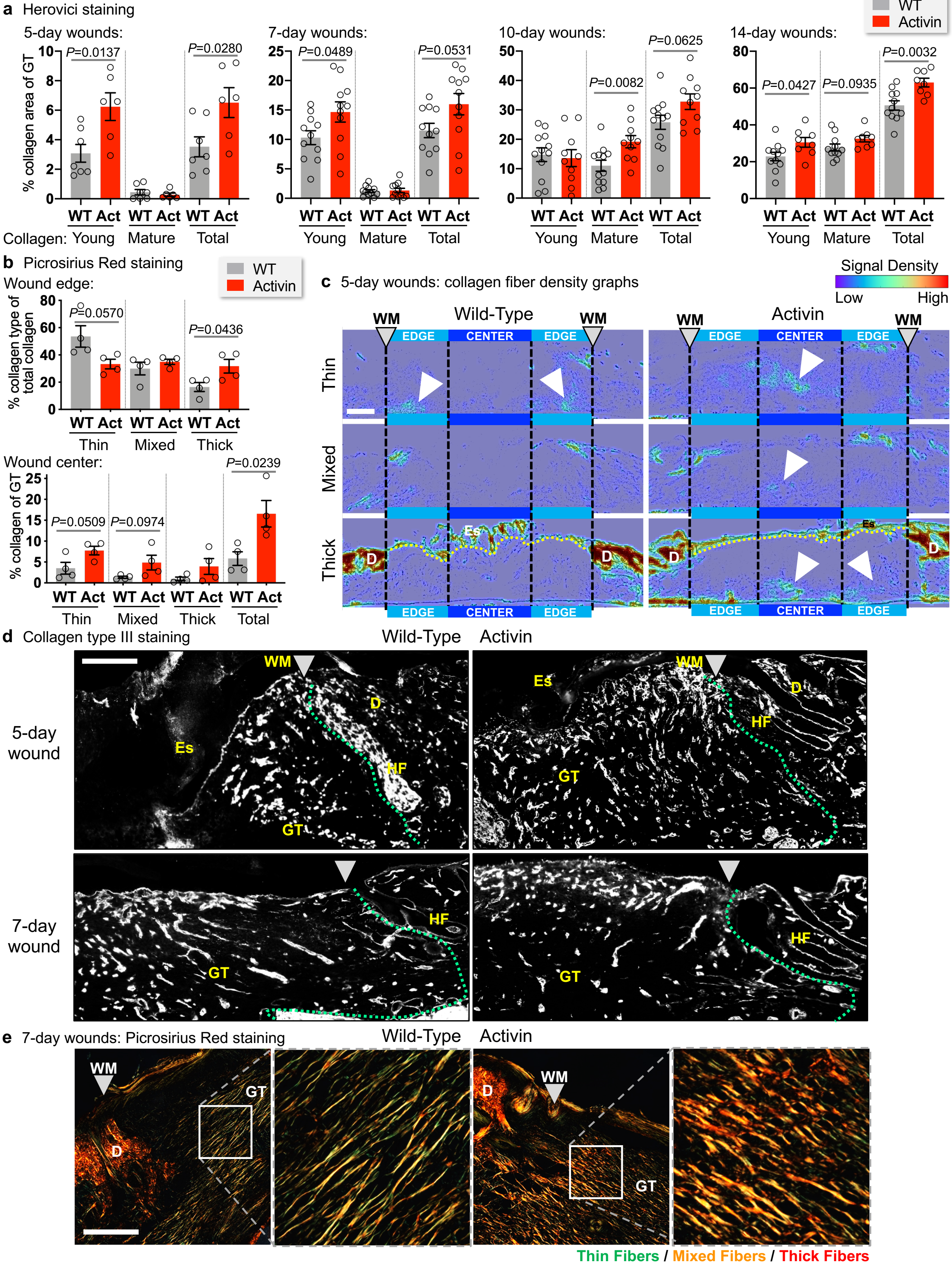

Supplementary Fig. 1: Activin promotes wound collagen deposition and maturation

(a) Comparison of young and mature collagen in 5-, 7-, 10- and 14-day wounds between Act *vs* WT mice based on Herovici staining, showing individual data points and *P*-values for comparisons illustrated in Fig. 1c. n=7,11,12,11 biological replicates for WT, n=6,11,10,8 for Act mice, for 5-, 7-, 10-, or 14-day wounds, respectively.

(b) Comparison of thin, mixed and thick collagen fibers in 5-day wound edges (top) and centers (bottom) between Act *vs* WT mice based on Picrosirius Red staining, showing individual data points and *P*-values for comparisons illustrated in Fig. 1f. n=4 biological replicates for WT and Act mice.

(c) Representative collagen fiber density graphs from Picrosirius Red-stained 5-day wounds of WT and Act mice, separated into thin (green), mixed (yellow) and thick (red) channels, and segmented into wound edge and center regions; white triangles point to areas of relatively high collagen density. Yellow dotted line represents extent of hyperproliferative epithelium. This analysis was performed on n=5 biological replicates with similar results.

(d) Representative photomicrographs of collagen III-stained sections of 5-day and 7-day wounds from WT and Act mice. Green dotted line represents border between dermis and granulation tissue. Staining was performed on n=5 biological replicates for 5-day wounds and n=3 for 7-day wounds with similar results.

(e) Representative photomicrographs of Picrosirius Red-stained sections of 7-day wounds from WT and Act mice, with insets showing greater detail of collagen fibers. This staining was performed on n=3 biological replicates with similar results.

Gray triangles represent wound margins (WM); HE, hyperproliferative epithelium; HF, hair follicle; D, dermis; Es, eschar, GT, granulation tissue. Scale bars: 250 μm. Graphs show mean

± SEM and *P* values; two-tailed Student *t* test for Act *vs* WT at each time-point for each type of collagen fiber.

Source data are provided as a Source Data file (Supplementary Figure 1).

Supplementary Figure 2

a PCA plot of RNA-seq samples

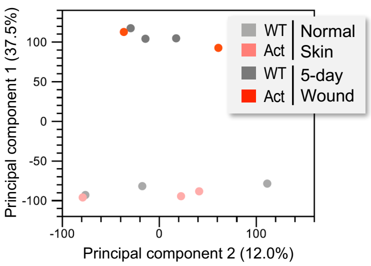

b Enrichment of genes in cell types (ImmGen MyGeneSet)

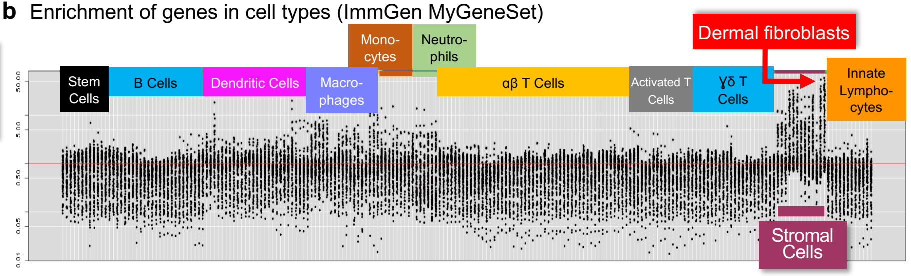

c Additional DEGs and other genes of interest

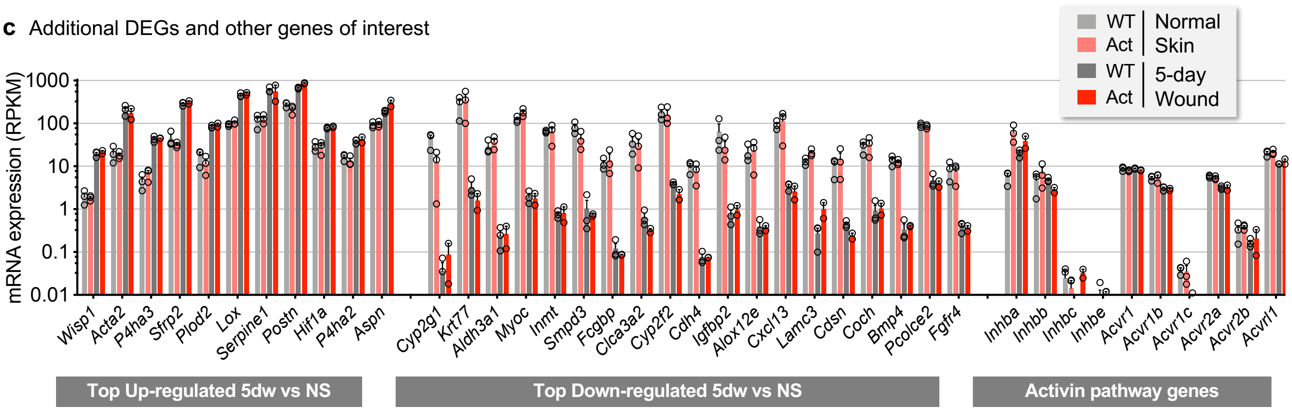

d PCA plot of 5dw FB samples

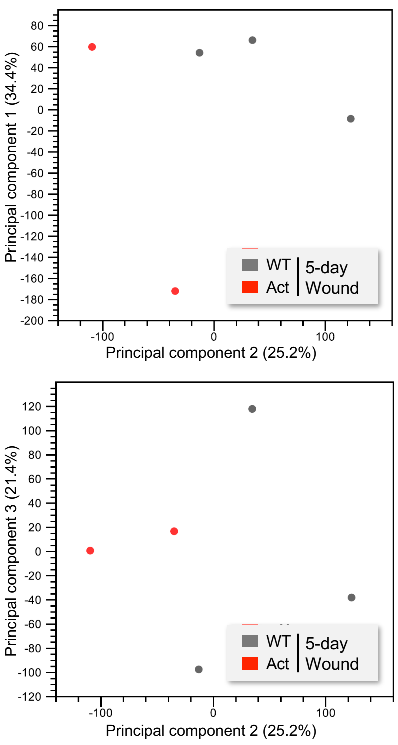

e Top Genes in Act vs WT NS FBs
(Max RPKM>5, $\log_2$FC>1.5, P-value<0.05)

Gene	$\log_2$FC	P-value	Avg RPKM WT	Avg RPKM Act
Inhba	11.49	4.15E-17	5.69	62.19
Lars2	9.94	9.76E-05	58.74	495.18
Rpph1	7.60	4.60E-03	0.65	5.16
Stfa3	6.88	1.45E-03	1.29	9.05
Krt6a	5.71	1.44E-04	8.63	46.76
Serpinb12	5.51	6.46E-04	1.14	6.39
Sprr1b	3.65	1.59E-03	14.86	50.84
Krt1	3.51	9.06E-05	120.45	395.55
Npl	2.81	4.42E-03	3.47	9.22
Aqp3	2.47	1.03E-02	26.04	60.06
Hmgcs2	2.17	3.29E-02	4.78	9.61
Pla2g2f	2.14	3.17E-02	13.52	27.09
Ifit3	2.03	6.20E-03	38.27	70.38
Ifit3b	2.02	2.78E-02	7.36	13.72
Calml3	1.95	3.50E-02	7.09	12.84
Calm4	1.91	4.29E-02	58.64	104.51

f Top Genes in Act vs WT 5dw FBs
(Max RPKM>5, $\log_2$FC>1.0, P-value<0.10)

Gene	$\log_2$FC	P-value	Avg RPKM WT	Avg RPKM Act
Slc25a44	1.96	3.71E-03	20.08	38.91
Angptl1	1.95	8.04E-03	13.92	26.93
Inhba	1.93	1.56E-02	20.12	38.21
Ptn	1.78	2.89E-03	22.30	39.14
H19	1.74	2.38E-06	260.63	445.78
Aspn	1.59	1.07E-03	188.66	296.42
C1qtnf3	1.56	2.07E-02	84.70	130.55
Marcks	1.48	1.77E-02	50.45	74.08
Itgbl1	1.46	4.02E-02	28.41	41.01
Rpl21	1.37	8.55E-02	29.54	39.84
Adamts4	1.36	2.59E-02	53.94	72.22
Dpysl3	1.35	6.68E-02	35.65	47.58
Ogn	1.35	8.28E-02	160.85	214.63
Postn	1.31	5.33E-02	664.97	859.71
Csrp2	1.24	3.57E-02	122.82	149.81
Fstl1	1.16	5.54E-02	862.79	989.85

g Enrichment Analysis

Down-regulated (189 genes):

GO Terms	FDR
regulation of insulin-like growth factor receptor signaling pathway	3.81E-04
extracellular matrix organization	3.81E-04
skin development	1.31E-03
cellular protein metabolic process	1.31E-03
cell-cell junction organization	1.82E-02
negative regulation of phosphorylation	1.90E-02
cell-cell junction assembly	2.17E-02
epidermis development	2.18E-02
negative regulation of endopeptidase activity	2.22E-02
negative regulation of cellular catabolic process	2.76E-02

h Leading Edge Analysis: co-enriched genes with 5-day wound FB signature and wound myofibroblast gene sets

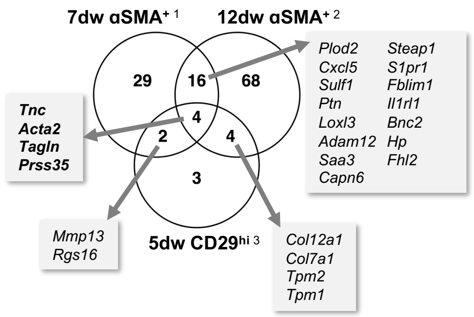

Supplementary Fig. 2: Wound fibroblasts exhibit a distinct transcriptional signature

(a) Principal component analysis (PCA) plot of all RNA-sequenced samples.

(b) Cell type enrichment via the ImmGen MyGeneSet web tool using overlapping genes from Fig. 2c, highlighting dermal fibroblasts and other stromal cells.

(c) Graphs showing absolute expression (in RPKM) of selected genes. Left*:* Additional top up-regulated genes (in 5dw *vs* NS comparisons). Middle*:* Top down-regulated genes (in 5dw *vs* NS comparisons). Right*:* Activin pathway genes, including receptors and ligands. Graph shows mean ± SEM; n=3 biological replicates for WT_NS, WT_5dw, Act_NS, n=2 for Act_5dw.

(d) PCA plots of RNA-sequencing data from 5dw fibroblast samples along PC1/2 (top) and PC3/2 (bottom).

(e) Table of top up-regulated genes in baseline comparison of Act vs WT NS fibroblasts (RPKM>5, $Log_2FC>1.5$, P-value<0.05).

(f) Table of top up-regulated genes in baseline comparison of Act vs WT 5dw fibroblasts (RPKM>5, $Log_2FC>1.0$, P-value<0.10).

(g) Shared down-regulated DEGs from Fig. 2e (FDR<0.05, $Log_2FC<-1$) were subjected to functional enrichment analysis using enrichR. Top 10 Gene Ontology (GO) Biological Processes are shown with respective FDR values.

(h) Leading edge analyses of GSEA from Fig. 2h, showing co-enriched genes in 5-day wound fibroblasts and wound myofibroblast datasets [1-3].

Source data are provided as a Source Data file (Supplementary Figure 2).

Supplementary Figure 3

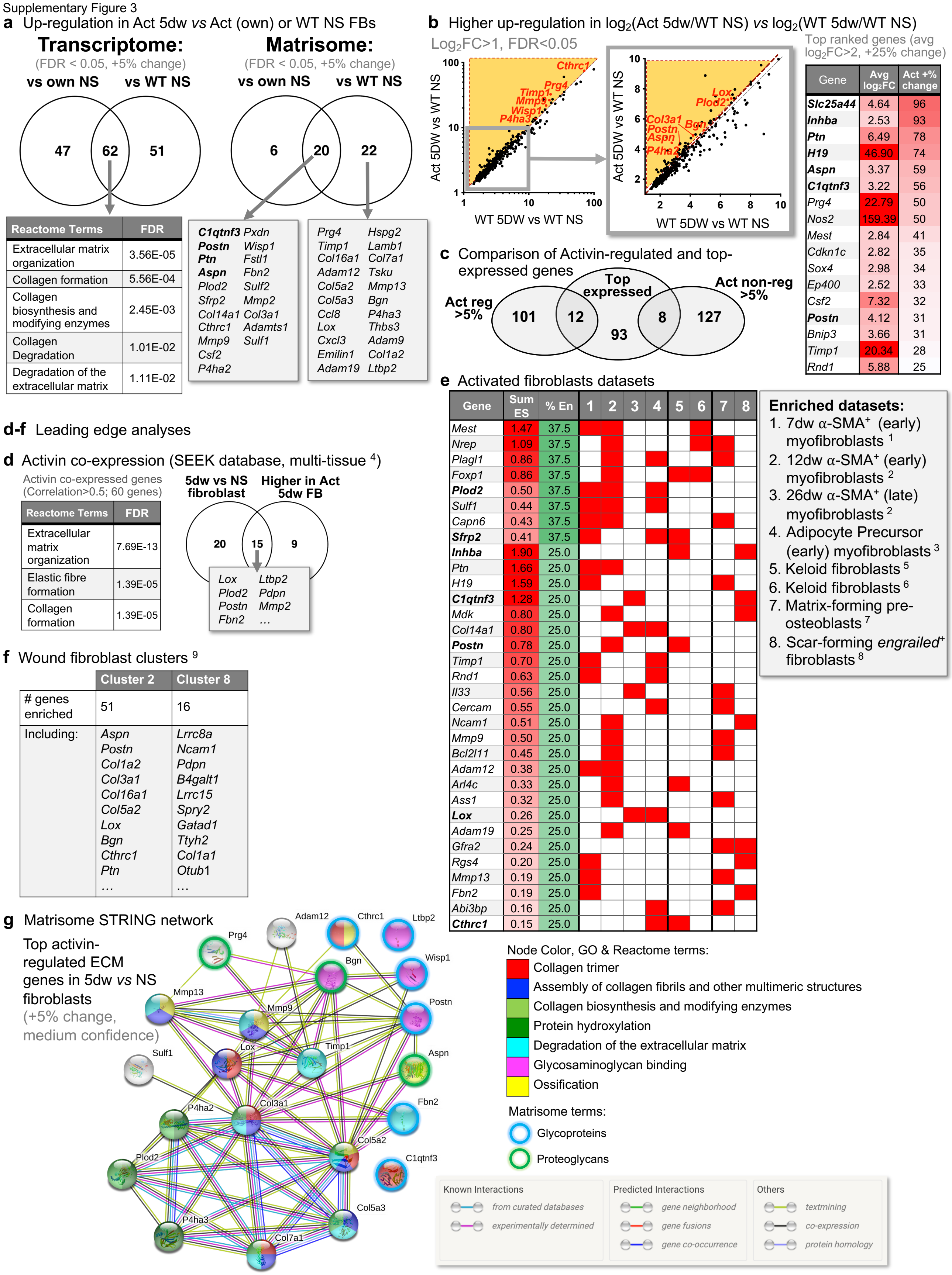

Supplementary Fig. 3: Activin induces a pro-fibrotic gene expression signature in fibroblasts

(a) Venn diagram showing genes up-regulated in fibroblasts of 5dw of Act *vs* NS mice relative to NS of either WT or Act mice, at the complete transcriptome (left) and the matrisome (right) levels. Lists were generated by ranking significantly expressed genes (FDR<0.05) according to >1.05 in the following two ratios: 1) "*vs* own NS" = $\log_2$(Act 5dw/Act NS)/$\log_2$(WT 5dw/WT NS), and 2) "*vs* WT NS" = $\log_2$(Act 5dw/WT NS)/$\log_2$(WT 5dw/WT NS). Bottom left: Functional enrichment according to Reactome is shown for overlapping transcriptome genes. Bottom right: Overlap and "*vs* WT NS" matrisome genes are listed; bold refers to presence in baseline up-regulation in Act *vs* WT 5dw (Supplementary Fig. 2f).

(b) Selected genes that are more strongly up-regulated in $\log_2$(Act 5dw/WT NS) *vs* $\log_2$(WT 5dw/WT NS). Left: Scatter plot (logarithmic scale) of DEGs showing selected genes (red) in the areas of >5% change (orange); inset (linear scale) shows genes in the $\mathrm{Log_2FC}$ 1-10 range. Right: Top ranked genes are listed, with average $\mathrm{Log_2FC}>2$ and >25% increase in activin 5dw fibroblasts; genes highlighted in bold are also up-regulated in the baseline comparison of 5dw Act *vs* WT (Supplementary Fig. 2f).

(c) Venn diagram showing overlap of fibroblast top expressed genes with activin-regulated and activin non-regulated genes.

(d-f) Leading edge analyses of GSEA from Fig. 3a-d, showing comparisons of genes co-enriched in activin-regulated genes and various gene sets.

(d) Genes significantly co-expressed with *INHBA*/activin were extracted from SEEK database of multi-tissue datasets (Spearman correlation>0.50) [4]. Left: top three enriched Reactome

pathways are shown. Right: co-enriched genes in 5dw wound fibroblast signature and activin-regulated genes are compared, and some ECM-related overlap genes are listed.

(e) Co-enriched activin-regulated genes in activated fibroblast gene sets [1-3,5-8], described by numbers 1-8 and genes ranked by percent dataset enrichment (% En, green) and the summed Enrichment Score (Sum ES, red). Genes highlighted in bold are mentioned in the text.

(f) Co-enriched activin-regulated genes in gene sets of wound fibroblast clusters No. 2 and 8 identified by single-cell RNA-seq [9].

(g) The top activin-regulated ECM genes (more than 5% change *vs* WT) were subjected to STRING analysis, and the resulting color-annotated gene-gene interaction network is shown. Top right: legend for node colors referring to Gene Ontology (GO) and Reactome terms, and node outline colors referring to Matrisome categories. Bottom right: STRING-derived legend for node edges.

Source data are provided as a Source Data file (Supplementary Figure 3).

Supplementary Figure 4

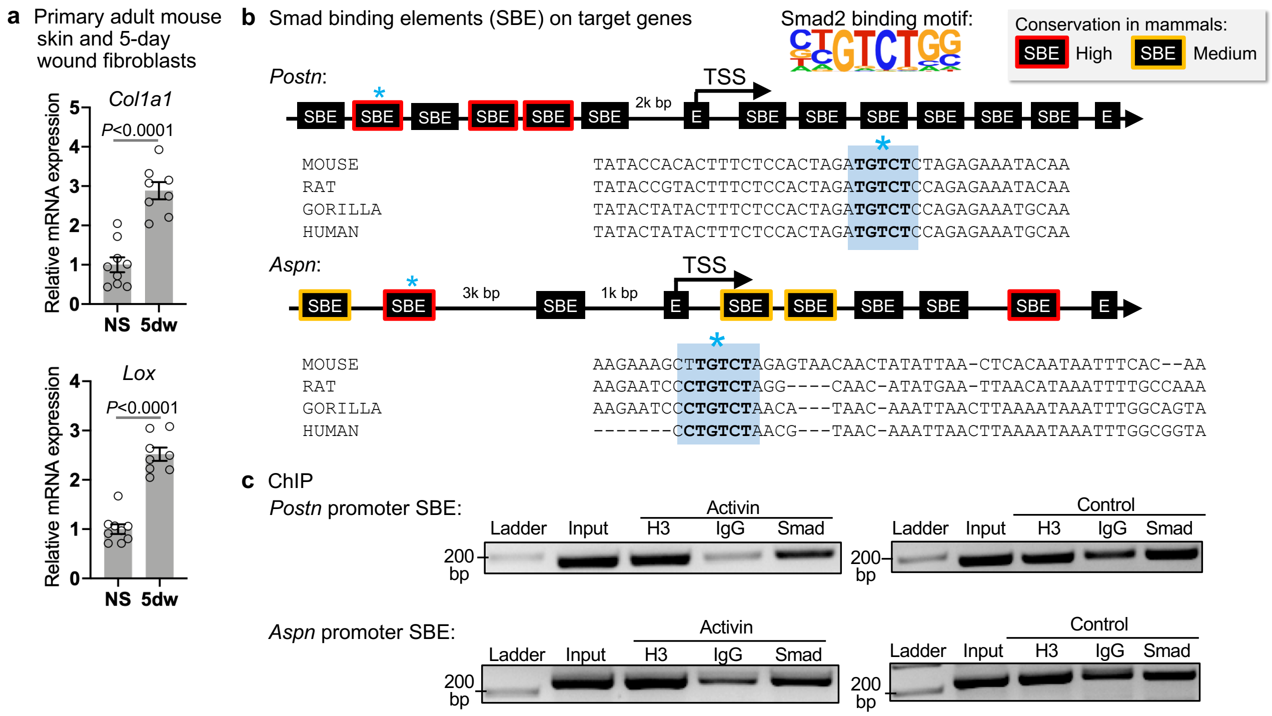

Supplementary Fig. 4: Activin is a direct regulator of ECM genes in fibroblasts

(a) Primary murine fibroblasts from normal skin (NS) and 5-day wounds (5dw) of 10 weeks-old WT mice were grown 80-90% confluency and gene expression was analyzed by qRT-PCR relative to *Rps29*. Graphs show mean ± SEM and *P* values; n=9 biological replicates for NS, n=8 for 5dw; two-tailed Student *t* test.

(b) Diagram showing location and conservation level of Smad-binding elements (SBE) in the promoter/first intron regions of *Postn* and *Aspn*. Blue asterisk refers to the sequence shown below, with the SBE highlighted and corresponding sequence similarity between the mouse, rat, gorilla and human SBE. TSS: transcription start site; E: exon.

(c) Immortalized mouse fibroblasts were treated with recombinant activin A (20 ng/ml) or vehicle (Control) for 6 h, and chromatin immunoprecipitation (ChIP) was performed using rabbit anti-histone H3 antibody (H3), normal rabbit IgG (IgG), or rabbit anti-SMAD2/3 antibody (Smad). The bound DNA was amplified using primers spanning conserved Smad binding

elements (SBEs) in the promoter regions of *Postn* and Aspn. Agarose gels are shown, with the 100bp DNA ladder (size marker) and respective inputs. This experiment was performed three times with similar results (see Fig. 4c for another replicate experiment).

Source data are provided as a Source Data file (Supplementary Figure 4).

Supplementary Figure 5

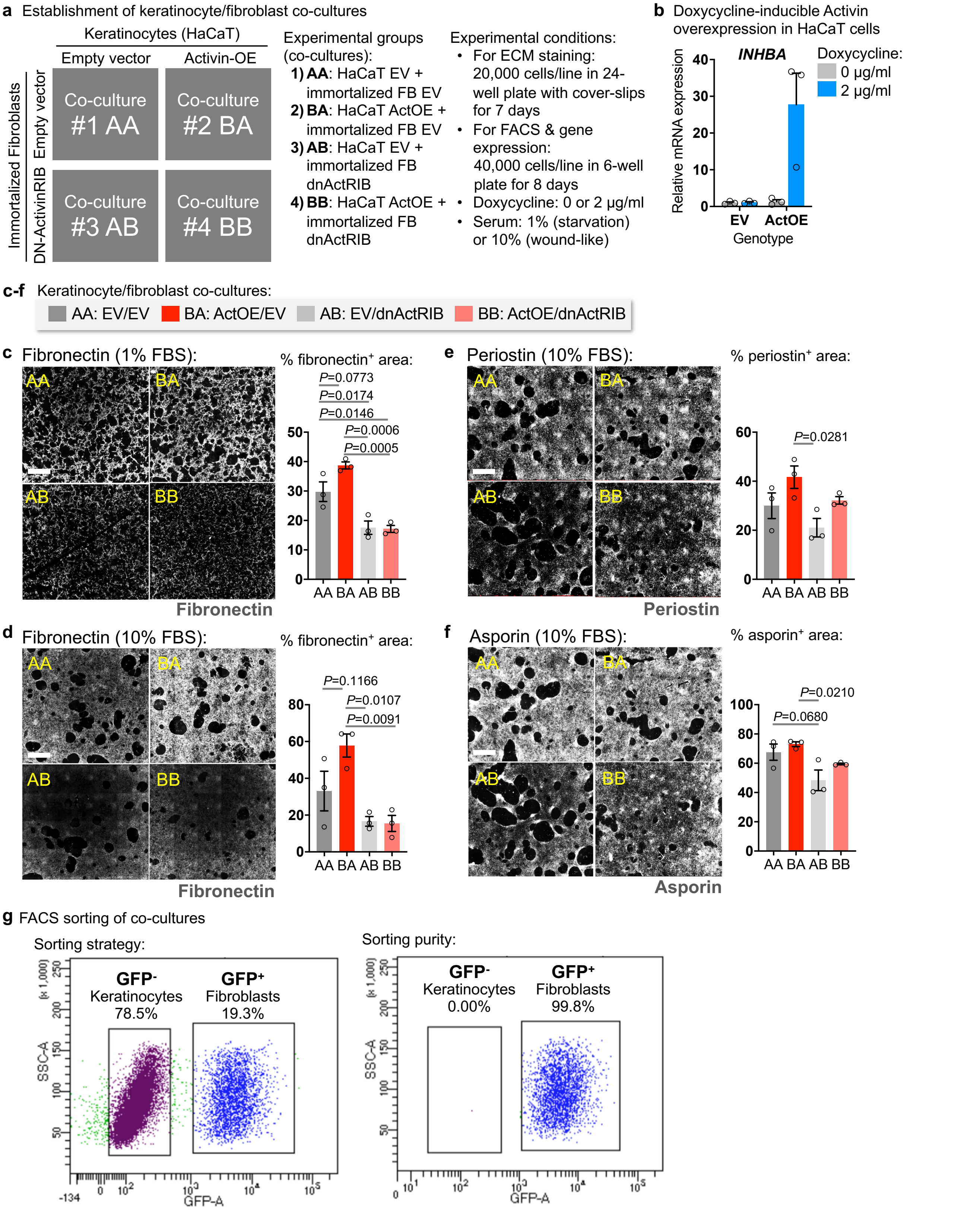

Supplementary Fig. 5: Activin regulates ECM deposition by fibroblasts

(a) Schematic representation of the experimental setup for keratinocyte (HaCaT) and fibroblast (immortalized) co-culture experiments, with labeling of the four experimental groups and list of experimental conditions.

(b) Empty vector (EV)-transduced cells or activin-overexpressing (ActOE) HaCaT cells were treated for 24 h with 2 µg/ml doxycycline (Dox) or vehicle and expression of *INHBA* was determined by qRT-PCR relative to *RPL27*. Graph shows mean ± SEM; n=3 biological replicates.

(c-f) HaCaT keratinocytes transduced with lentiviruses allowing Dox-inducible overexpression of *INHBA* (ActOE) or control viruses (empty vector; EV) were co-cultured for 7d with murine immortalized fibroblasts (GFP-expressing) transduced with lentiviruses allowing Dox-inducible expression of dnActRIB or control viruses (EV). Representative photomicrographs are shown for **(c)** fibronectin staining for cells cultured in the presence of 1% FBS, **(d)** fibronectin staining for cells cultured in the presence of 10% FBS, **(e)** periostin staining for cells cultured in the presence of 10% FBS, and **(f)** asporin staining for cells cultured in the presence of 10% FBS. Bar graphs show quantification of percentage of stained area on whole co-culture coverslips. The experiment was repeated twice with similar results. Scale bars: 1000 μm. Graphs show mean ± SEM and *P* values; n=3 biological replicates; one-way ANOVA and Tukey's multiple comparison post-hoc tests.

(g) Representative gating strategy for sorting of GFP^+ fibroblasts from GFP^- keratinocytes in the co-culture experiments. The relative percentage of each population is indicated. Right panel: the sorted GFP^+ sample was re-analyzed to evaluate the purity of the sorted cells. Source data are provided as a Source Data file (Supplementary Figure 5).

Supplementary Figure 6

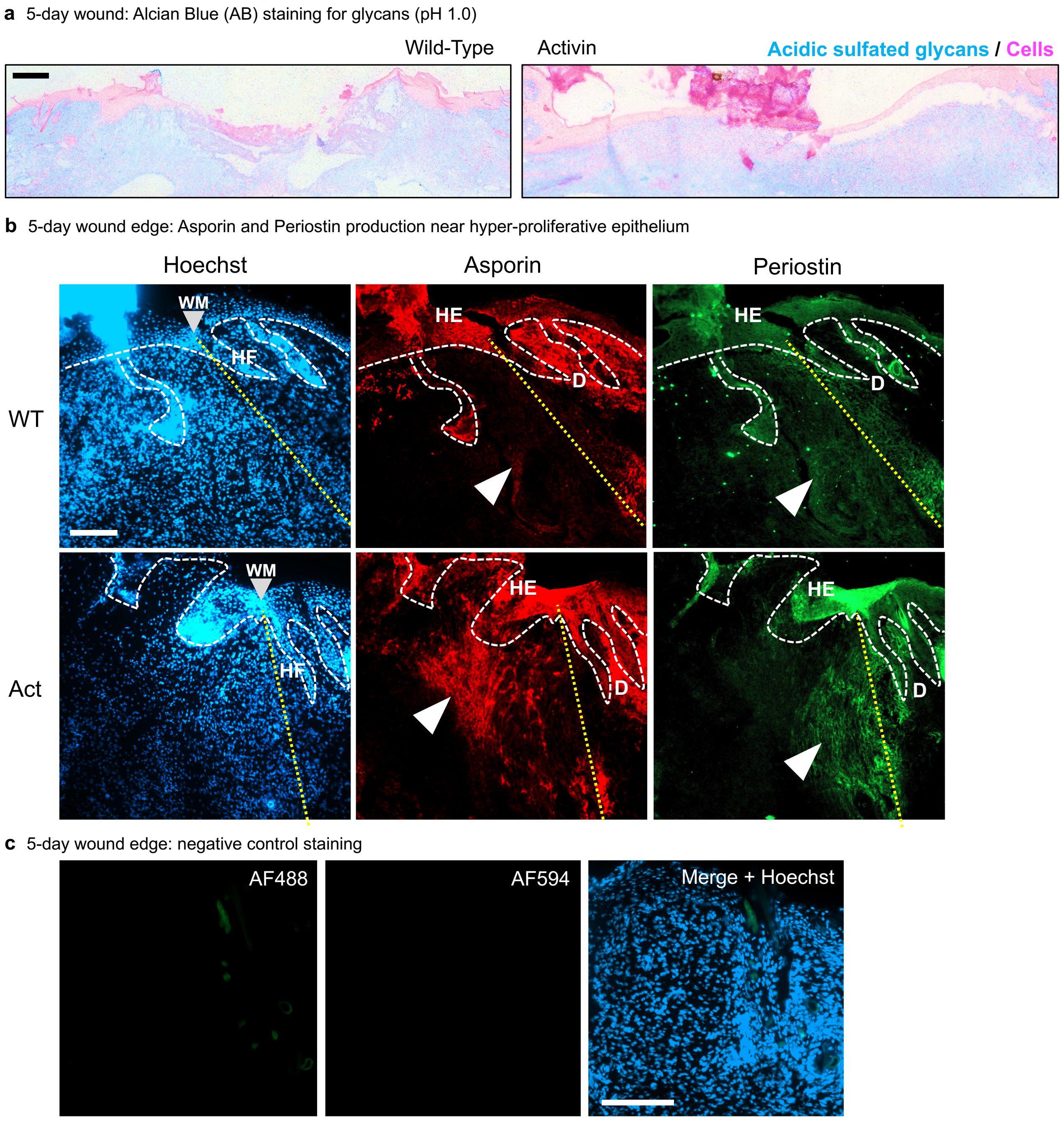

Supplementary Fig. 6: Activin promotes deposition of glycoproteins and proteoglycans in wounds

(a) Representative photomicrographs of Alcian Blue (pH 1)-stained sections from 5-day wounds showing areas of acidic-sulfated (blue) glycans in the wound centers of Act *vs* WT mice.

(b) Representative photomicrographs of asporin (red) and periostin (green) co-staining at the edge of 5d wounds. Nuclei were counterstained with Hoechst (blue). White arrowheads indicate areas of robust ECM staining at the wound edge; gray arrowheads indicate wound margins (WM). HE, hyperproliferative epithelium; HF, hair follicle; D, dermis. White dotted lines indicate the border between the dermis/granulation tissue and the epidermis or wound epithelium; yellow dotted lines indicate the dermis/granulation tissue border.

(c) Representative photomicrographs of negative controls (no primary antibody) for the immunofluorescence stainings shown in Fig. 6b-e and Supplementary Fig. 6b.

These stainings were performed on n=5 biological replicates with similar results.

Scale bars: 200 μm.

Supplementary Figure 7

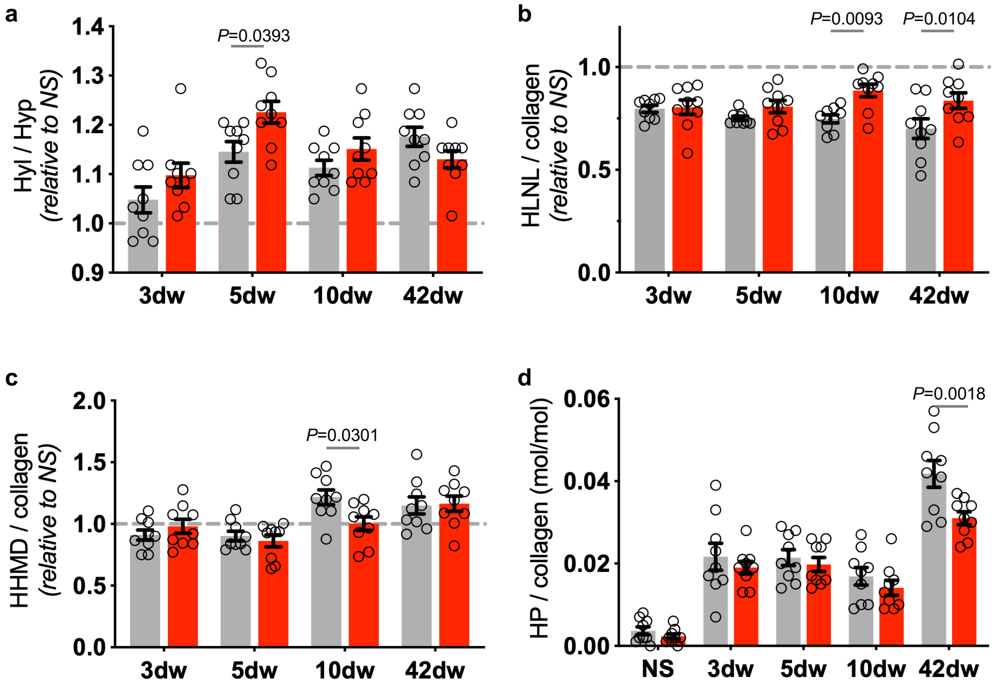

Supplementary Fig. 7: Activin promotes collagen maturation in healing wounds

Biochemical characterization of collagen using whole lysate of normal skin (NS) and wounds at days 3, 5, 10 and 42 post-injury.

(a) Hyl/Hyp ratio relative to NS (gray dashed line).

(b) HLNL/collagen ratio relative to NS (gray dashed line).

(c) HHMD/collagen ratio relative to NS (gray dashed line).

(d) HP/collagen per sample.

Graphs show mean ± SEM and *P* values; n=9 biological replicates for WT and Act at all time-points; two-way ANOVA and Bonferroni's multiple comparison post-hoc tests.

Lys, lysine; Hyl, hydroxylysine; Hyp, hydroxyproline; Lys^{ald}, lysine aldehyde; Hyl^{ald}, hydroxylysine aldehyde; DHLNL, dihydroxylysinonorleucine; HHMD, histidinohydroxymerodesmosine; HLNL, hydroxylysinonorleucine; HP, hydroxylysylpyridinoline.

Source data are provided as a Source Data file (Supplementary Figure 7).

Supplementary Figure 8

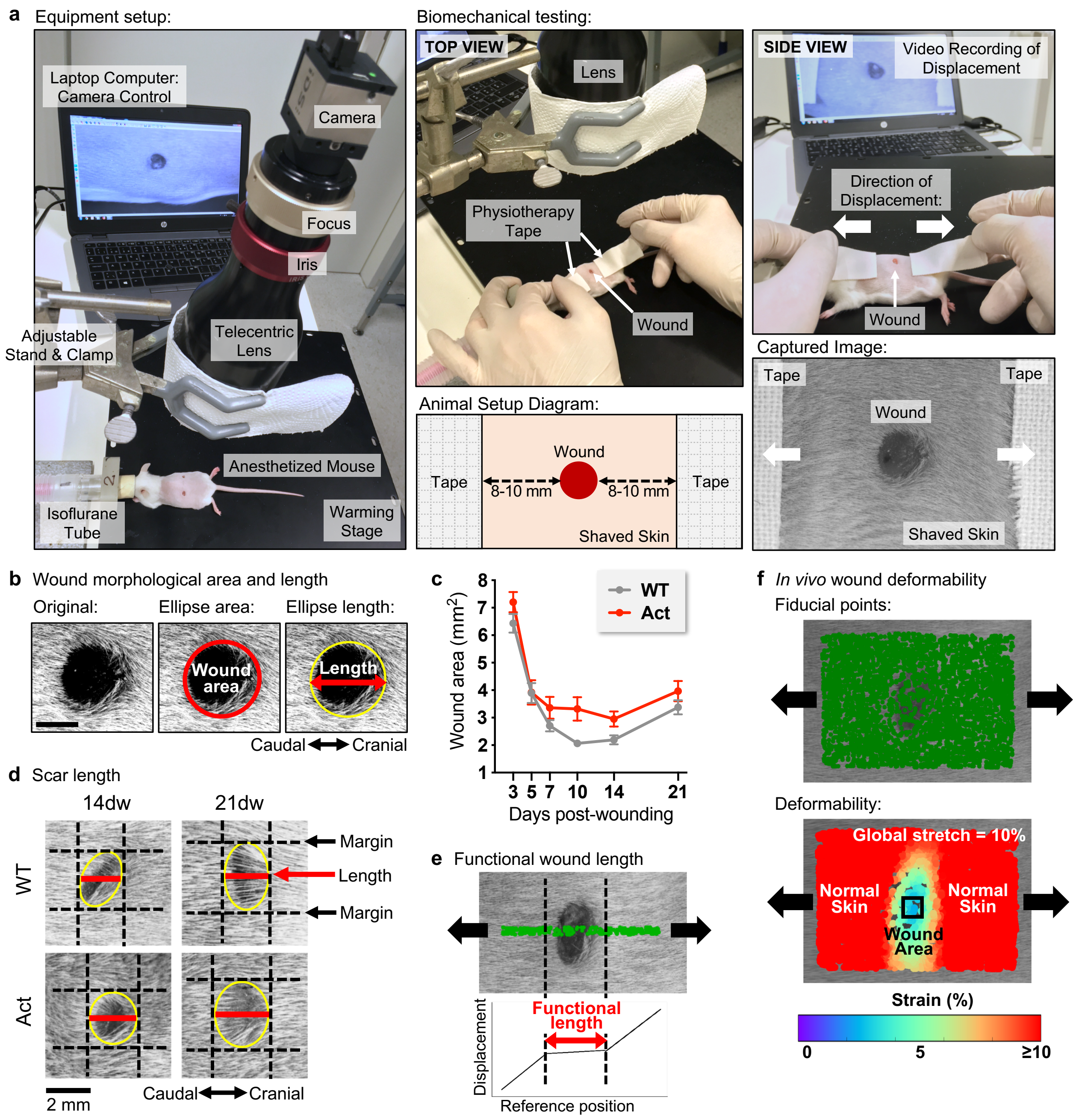

Supplementary Fig. 8: Biomechanical analyses of excisional skin wounds

(a) Annotated photos and diagrams of the non-invasive testing setup developed to monitor the morphological and biomechanical properties of murine wounds. Equipment setup: The digital camera and telecentric lens (including iris and focus adjustments) are held directly above the operating stage using an adjustable stand and clamp. A laptop computer controls the camera

operation via a software interface, allowing for reliable contactless monitoring of the wound region. The operating stage is warmed to 37°C to ensure comfort of the animal under isoflurane anaesthesia. Biomechanical testing: The top and side views show the placement of the medical-grade tape on both sides of the wound, which is manually pulled by the operator to extend the tissue approximately along the cranial-caudal directions (white arrows), while the camera monitors the deforming tissue. Animal Setup Diagram: Schematic representation of the tape placement on shaved skin on both sides of the wound. Captured Image: Representative still image from the video recording, showing tape placement on shaved skin on both sides of the wound and the direction of applied displacement (white arrows).

(b) Diagram showing the image processing procedure for quantification of wound morphological parameters, from the original image (left) to the determination of the area (middle) and length along the cranial-caudal direction (right) of an ellipse that is fitted to the visible wound. Scale bar: 2 mm.

(c) Quantification of wound areas according to image processing (see Supplementary Fig. 8b middle). Graph shows mean ± SEM; n=24,10,12,14,16,12 biological replicates for WT and n=22,6,10,8,12,8 for Act mice in 3-, 5-, 7-, 10-, 14-, or 21-day wounds, respectively.

(d) Diagram showing the image analysis procedure for quantification of visible scar length on days 14 and 21 post-injury. Scale bar: 2 mm.

(e) Diagram showing the image analysis procedure for quantification of the functional wound length, by measuring the gradients of tissue displacement along a narrow strip including both wounded and unwounded skin (green fiducial points), and roughly aligned with the direction of the applied displacement (black arrows).

(f) Diagram showing the procedure to quantify the local wound deformability under a global applied displacement (black arrows). Top: Still image from recorded video, overlaid with fiducial points (green) defining the region of interest for deformation analysis. Bottom: Color-map displaying the results for a 10% overall deformation level as well as the region (black box) used for quantitative comparison of wound deformability across time points and among genotypes.

Source data are provided as a Source Data file (Supplementary Figure 8).

Supplementary Figure 9

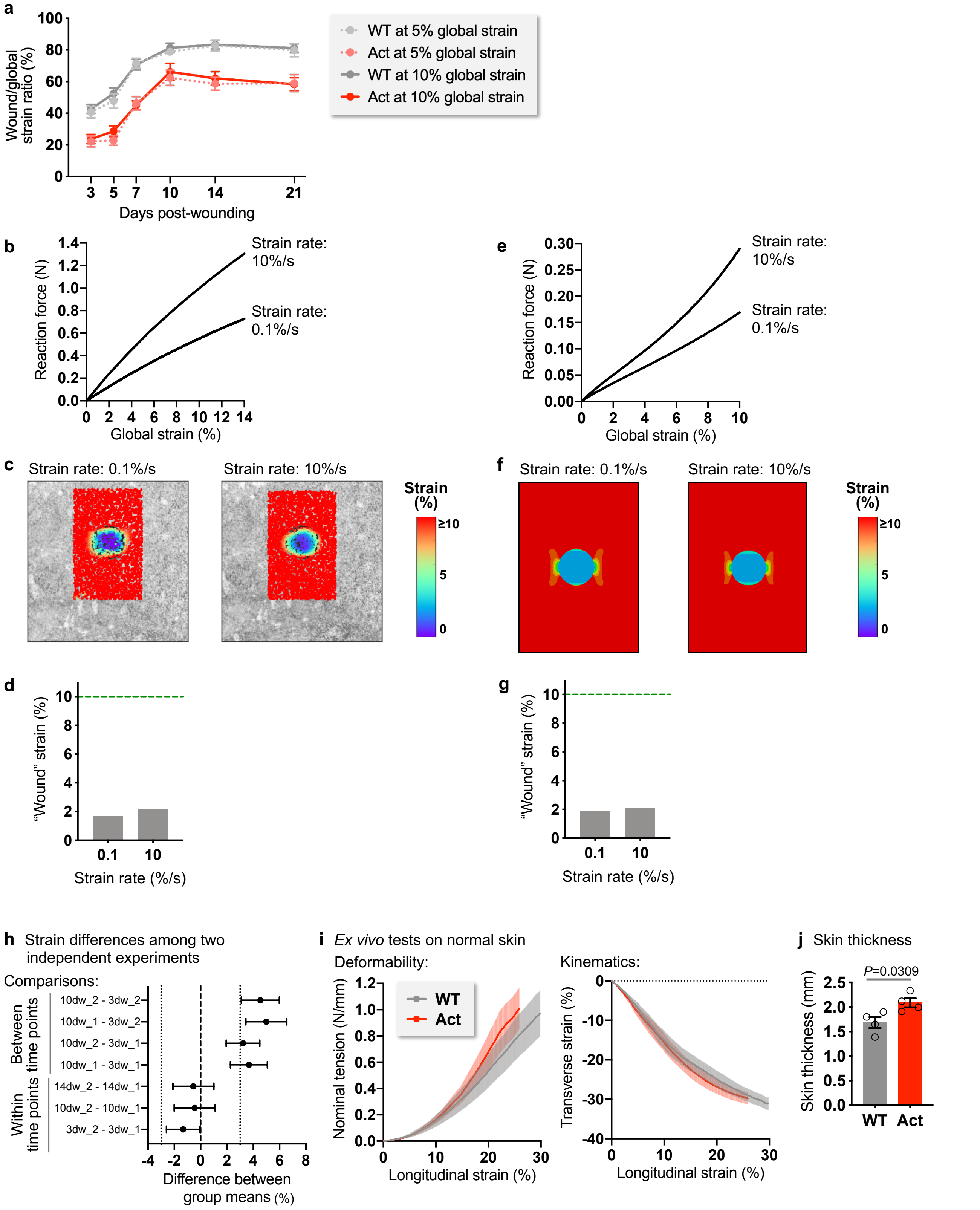

Supplementary Fig. 9: Validation of the biomechanical analysis technique

(a) Influence of the global strain value chosen for analysis of wound deformability. Reducing the global strain from 10% to 5% did not significantly alter the measured progression of the biomechanical properties. Graph shows mean ± SEM; n=24,10,12,14,16,12 biological replicates for WT and n=22,6,10,8,12,8 for Act mice in 3-, 5-, 7-, 10-, 14-, or 21-day wounds, respectively.

(b-d) Validation of surface elastography method on a synthetic model system with visco-elastic mechanical behavior. **(b)** The visco-elastic response of the specimen is indicated by the force increase associated with an increase in the applied strain rate. **(c)** The elastograms obtained at strain rates of 0.1%/s and 10%/s are shown. **(d)** The average strain in the center of the stiff inclusion is shown. n=1 experiment.

(e-g) Finite-element simulations of wounded skin with elongation up to 10% global strain. **(e)** The visco-elastic response of the simulation is indicated by the force increase associated with an increase in the applied strain rate. **(f)** The strain patterns obtained at strain rates of 0.1%/s and 10%/s are shown. **(g)** Corresponding quantification of the strain within the stiff inclusion is shown. n=1 experiment.

(h) Strain differences among independent experiments performed on WT mice at d3, d10, and d14 (underscore number indicates independent group) between time-points (top) and within time-points (bottom). Mean values ± 90% confidence intervals by one-way ANOVA with Bonferroni's post-hoc tests are shown. Dotted lines at -3% and +3% indicate bounds for significance for equivalence tests (at 95% confidence); dashed line at 0% indicates threshold for significance for statistical difference (at 95% confidence). n=6 biological replicates for 10dw_1 and 14dw_1; n=8 for 3dw_2, 10dw_2, 14dw_2; n=14 for 3dw_1.

(i) Results of uniaxial tensile tests on unwounded skin specimens from WT and Act mice displaying nonlinear tension-deformation and kinematics curves. n=4 biological replicates.

(j) Thickness of unwounded skin from WT and Act mice. Graphs shows mean ± SEM and *P* values; n=4 biological replicates; two-tailed Student *t* test.

Source data are provided as a Source Data file (Supplementary Figure 9).

Supplementary Table 1: Comparison of wound or scar parameters across time-points.

Geno-type	Comparison	Visible wound or scar length (Fig. 8b) 95% CI of difference (mm)	Visible wound or scar length (Fig. 8b) Adjusted p-value	Functional wound length (Fig. 8d) 95% CI of difference (mm)	Functional wound length (Fig. 8d) Adjusted p-value	Wound strain (Fig. 8g) 95% CI of difference (%)	Wound strain (Fig. 8g) Adjusted p-value
Wild-Type	D3 vs. D5	-0.026 to 0.885	0.0842	-0.768 to 0.913	>0.9999	-2.348 to 0.409	0.5629
	D3 vs. D7	0.338 to 1.194	**<0.0001**	0.479 to 2.058	**<0.0001**	-4.091 to -1.501	**<0.0001**
	D3 vs. D10	0.591 to 1.406	**<0.0001**	1.475 to 2.977	**<0.0001**	-5.093 to -2.630	**<0.0001**
	D3 vs. D14	0.711 to 1.492	**<0.0001**	1.436 to 2.877	**<0.0001**	-5.250 to -2.887	**<0.0001**
	D3 vs. D21	0.398 to 1.254	**<0.0001**	1.452 to 3.031	**<0.0001**	-5.143 to -2.554	**<0.0001**
	D5 vs. D7	-0.181 to 0.855	0.8137	0.241 to 2.152	**0.0040**	-3.395 to -0.259	**0.0101**
	D5 vs. D10	0.068 to 1.070	**0.0136**	1.230 to 3.078	**<0.0001**	-4.408 to -1.376	**<0.0001**
	D5 vs. D14	0.184 to 1.160	**0.0010**	1.185 to 2.985	**<0.0001**	-4.576 to -1.623	**<0.0001**
	D5 vs. D21	-0.121 to 0.915	0.3556	1.213 to 3.125	**<0.0001**	-4.447 to -1.311	**<0.0001**
	D7 vs. D10	-0.244 to 0.708	>0.9999	0.079 to 1.836	**0.0213**	-2.506 to 0.375	0.4323
	D7 vs. D14	-0.127 to 0.797	0.4829	0.036 to 1.741	**0.0339**	-2.671 to 0.126	0.1110
	D7 vs. D21	-0.434 to 0.554	>0.9999	0.061 to 1.884	**0.0266**	-2.548 to 0.443	0.5595
	D10 vs. D14	-0.340 to 0.546	>0.9999	-0.886 to 0.748	>0.9999	-1.547 to 1.133	>0.9999
	D10 vs. D21	-0.648 to 0.304	>0.9999	-0.863 to 0.893	>0.9999	-1.428 to 1.454	>0.9999
	D14 vs. D21	-0.737 to 0.187	>0.9999	-0.768 to 0.937	>0.9999	-1.178 to 1.619	>0.9999
Activin	D3 vs. D5	0.183 to 1.298	**0.0017**	-0.807 to 1.250	>0.9999	-2.175 to 1.199	>0.9999
	D3 vs. D7	0.357 to 1.280	**<0.0001**	0.232 to 1.935	**0.0032**	-3.589 to -0.691	**0.0003**
	D3 vs. D10	0.462 to 1.462	**<0.0001**	1.196 to 3.040	**<0.0001**	-5.746 to -2.722	**<0.0001**
	D3 vs. D14	0.586 to 1.455	**<0.0001**	1.118 to 2.721	**<0.0001**	-5.144 to -2.515	**<0.0001**
	D3 vs. D21	0.297 to 1.297	**<0.0001**	1.346 to 3.190	**<0.0001**	-4.993 to -1.620	**<0.0001**
	D5 vs. D7	-0.547 to 0.703	>0.9999	-0.291 to 2.015	0.4074	-3.582 to 0.278	0.1750
	D5 vs. D10	-0.433 to 0.875	>0.9999	0.691 to 3.102	**<0.0001**	-5.724 to -1.768	**<0.0001**
	D5 vs. D14	-0.325 to 0.885	>0.9999	0.581 to 2.814	**0.0002**	-5.173 to -1.511	**<0.0001**
	D5 vs. D21	-0.598 to 0.710	>0.9999	0.841 to 3.252	**<0.0001**	-4.933 to -0.704	**0.0017**
	D7 vs. D10	-0.431 to 0.718	>0.9999	-0.025 to 2.093	0.0619	-3.874 to -0.315	**0.0089**
	D7 vs. D14	-0.316 to 0.720	>0.9999	-0.120 to 1.791	0.1506	-3.305 to -0.075	**0.0324**
	D7 vs. D21	-0.596 to 0.553	>0.9999	0.125 to 2.243	**0.0161**	-3.097 to 0.763	>0.9999
	D10 vs. D14	-0.494 to 0.611	>0.9999	-1.218 to 0.820	>0.9999	-1.267 to 2.076	>0.9999
	D10 vs. D21	-0.770 to 0.440	>0.9999	-0.966 to 1.266	>0.9999	-1.050 to 2.905	>0.9999
	D14 vs. D21	-0.776 to 0.329	>0.9999	-0.670 to 1.368	>0.9999	-1.308 to 2.354	>0.9999

Statistical comparisons of wound parameters across time-points; two-way ANOVA and Bonferroni's multiple comparison post-hoc tests. n=24, 10, 12, 14, 16, or 12 for WT and n=22, 6, 10, 8, 12, or 8 for Act mice in 3-, 5-, 7-, 10-, 14-, or 21-day wounds, respectively. Bold refers to adjusted p-values<0.05.

Supplementary Table 2: Sequences of primers used for qRT-PCR.

Gene	Forward Primer (5'-3')	Reverse Primer (5'-3')
Rps29	GGT CAC CAG CAG CTC TAC TG	GTC CAA CTT AAT GAA GCC TAT GTC C
RPL27	TCA CCT AAT GCC CAC AAG GTA	CCA CTT GTT CTT GCC TGT CTT
ActRIB	GGG TGG GGA CCA AAC GAT AC	GAG GGC ATA GAT GTC GGC AC
dnActRIB	TCA TCG TCT TCC TGG TCA TCA A	ATC AGT TTC TGT TCC CCA CCA C
Aspn	TCT CCT CTG ACA AGG TTG GAC	TTC AGT GCT GTG TGG GAA GG
Col1a1	TGT TCA GCT TTG ACC TCC GGC T	TCT CCC TTG GGT CCC TCG ACT
INHBA	CCT CGG AGA TCA TCA CGT TT	CCC TTT AAG CCC ACT TCC TC
Lox	ACT GCA CAC ACA CAG GGA TT	TGT AGC GAA TGT CAC AGC GT
Postn	AAG ACT GCT TCA GGG AGA CAC A	TCA GTG TGG TGG CTC TTA CA
Wisp1	AAT AGG AGT GTG TGC ACA GGT	CTC GCC ATT GGT GTA GCG TA

Supplementary Table 3: Primer sequences for amplification of fragments for cloning.

Gene	Forward Primer (5'-3')	Reverse Primer (5'-3')
*INHBA**	CAC CAT GCC CTT GCT TTG	CTA TGA GCA CCC ACA CTC CTC C
*dnActRIB***	CAC CAT GGC GGA GTC GGC CGG AGC CTC	TTA CAG GTC TTC TTC AGA GAT CAG TTT CTG TTC

An in-frame *HA-tag or **MYC-tag sequence was fused in-frame at the 3' end of the coding sequence

Supplementary Table 4: Primer sequences for amplification of ChIP products.

Gene	Forward Primer (5'-3')	Reverse Primer (5'-3')
Postn promoter*	TGT GAC AGA CAT TTC CAG TGA	TGG GTC ATT CCC CTA TTG AG
Aspn promoter**	GCA TGA TGC ACA CTC ACA CA	CCT GTG GCC TTG AAA TTG TT

Product size: *186 bp, **228 bp

Supplementary References

1. Bergmeier V*, et al.* Identification of a myofibroblast-specific expression signature in skin wounds. *Matrix Biol* **65**, 59-74 (2018).

2. Plikus MV, *et al*. Regeneration of fat cells from myofibroblasts during wound healing. *Science* **355**, 748-752 (2017).

3. Shook BA, *et al*. Myofibroblast proliferation and heterogeneity are supported by macrophages during skin repair. *Science* **362**, (2018).

4. Zhu Q, *et al*. Targeted exploration and analysis of large cross-platform human transcriptomic compendia. *Nat Methods* **12**, 211-214, 213 p following 214 (2015).

5. Hahn JM, Glaser K, McFarland KL, Aronow BJ, Boyce ST, Supp DM. Keloid-derived keratinocytes exhibit an abnormal gene expression profile consistent with a distinct causal role in keloid pathology. *Wound Repair Regen* **21**, 530-544 (2013).

6. Smith JC, Boone BE, Opalenik SR, Williams SM, Russell SB. Gene profiling of keloid fibroblasts shows altered expression in multiple fibrosis-associated pathways. *J Invest Dermatol* **128**, 1298-1310 (2008).

7. Wu H, *et al*. Genomic occupancy of Runx2 with global expression profiling identifies a novel dimension to control of osteoblastogenesis. *Genome Biol* **15**, R52 (2014).

8. Rinkevich Y, *et al*. Skin fibrosis. Identification and isolation of a dermal lineage with intrinsic fibrogenic potential. *Science* **348**, aaa2151 (2015).

9. Guerrero-Juarez CF, *et al*. Single-cell analysis reveals fibroblast heterogeneity and myeloid-derived adipocyte progenitors in murine skin wounds. *Nat Commun* **10**, 650 (2019).
