# Supplementary material for: Activin-mediated alterations of the fibroblast transcriptome and matrisome control the biomechanical properties of skin wounds
Source: Nat Commun. 2020 May 25;11:2604. doi: 10.1038/s41467-020-16409-z (PMC7248062; doi:10.1038/s41467-020-16409-z)
Supplement: Supplementary file 2 — Reporting summary [file 41467_2020_16409_MOESM2_ESM.pdf]

nature research

Corresponding author(s): Sabine Werner, Edoardo Mazza

Last updated by author(s): Apr 14, 2020

Reporting Summary

Nature Research wishes to improve the reproducibility of the work that we publish. This form provides structure for consistency and transparency in reporting. For further information on Nature Research policies, seeAuthors & Referees and theEditorial Policy Checklist .

Statistics

For all statistical analyses, confirm that the following items are present in the figure legend, table legend, main text, or Methods section.

n/a	Confirmed	
☐	☒	The exact sample size (*n*) for each experimental group/condition, given as a discrete number and unit of measurement
☐	☒	A statement on whether measurements were taken from distinct samples or whether the same sample was measured repeatedly
☐	☒	The statistical test(s) used AND whether they are one- or two-sided<br>*Only common tests should be described solely by name; describe more complex techniques in the Methods section.*
☐	☒	A description of all covariates tested
☐	☒	A description of any assumptions or corrections, such as tests of normality and adjustment for multiple comparisons
☐	☒	A full description of the statistical parameters including central tendency (e.g. means) or other basic estimates (e.g. regression coefficient) AND variation (e.g. standard deviation) or associated estimates of uncertainty (e.g. confidence intervals)
☐	☒	For null hypothesis testing, the test statistic (e.g. *F*, *t*, *r*) with confidence intervals, effect sizes, degrees of freedom and *P* value noted<br>*Give P values as exact values whenever suitable.*
☒	☐	For Bayesian analysis, information on the choice of priors and Markov chain Monte Carlo settings
☒	☐	For hierarchical and complex designs, identification of the appropriate level for tests and full reporting of outcomes
☒	☐	Estimates of effect sizes (e.g. Cohen's *d*, Pearson's *r*), indicating how they were calculated

Our web collection on statistics for biologists contains articles on many of the points above.

Software and code

Policy information about availability of computer code

Data collection	No software was used.
Data analysis	Quantitative data from all experiments were organized in Microsoft Excel 2016, then transferred into Graphpad PRISM (ver 8) for statistical analyses and graph generation.<br>FACS: FACSDiva (ver 6) for analysis during acquisition and sorting; FlowJo (ver X) for data analysis.<br>RNA-seq: CLC Genomics Workbench (ver 12) for quality control and initial analysis; enrichR (ver 2019) for enrichment analysis; Ingenuity Pathway Analysis (ver 2018) for pathway analysis; GSEA (ver 3) for gene set enrichment analysis.<br>Histology: ImagePro Plus (ver 4.5) for color analysis of Herovici- and Picrosirius Red-stained sections; Fiji or Image J (ver 2019) for analysis of immunofluorescence sections.<br>Biomechanics: Image analysis for strain quantification was performed using a custom-written Python code that was previously presented (see, e.g. Hopf, et al. J Mech Behav Biomed Mater (2016)). The code builds on the freely-available OpenCV package, which provides a ready-to-use implementation the Lukas-Kanade optical flow algorithm to track the time-evolving location of fiducial points within a region of interest. Use of our custom-written code to quantify the strain field in wounded and unwounded tissues, as well as in elastomers, has been presented, e.g. in Pensalfini, et al. Acta Biomater (2018). The 2D inhomogeneous strain field within and around a wound is obtained from point tracking by using weighted least-square fitting to estimate the local strain at each fiducial point location.<br>Any state of the art software for digital image correlation could be applied to reconstruct the (homogeneous or inhomogeneous) strain field in the performed experiments and thus reproduce the presented results. Examples are the commercial software ARAMIS or Vic-Software, as well as the freely-available Ncorr (MATLAB-based) and py2DIC (Python-based). Finite element analysis simulations were performed using the commercial software Abaqus/Standard (ver. 6.14-1). The adopted material model and element formulation were previously published and validated for the simulation of the mechanical behavior of skin (see e.g. Weickenmeier, et al. Int J Numer Meth Bio (2014) and Weickenmeier, et al. Journal of Biomechanics (2015)).

For manuscripts utilizing custom algorithms or software that are central to the research but not yet described in published literature, software must be made available to editors/reviewers. We strongly encourage code deposition in a community repository (e.g. GitHub). See the Nature Research guidelines for submitting code & software for further information.

Data

Policy information about availability of data

All manuscripts must include a data availability statement. This statement should provide the following information, where applicable:

- Accession codes, unique identifiers, or web links for publicly available datasets
- A list of figures that have associated raw data
- A description of any restrictions on data availability

RNA sequencing data from skin and wound fibroblasts of WT and Act mice can be accessed via the Gene Expression Omnibus (GSE134789). Original source data from Figures 1, 2, 3, 4, 5, 7, 8 and Supplementary Figures 1, 2, 3, 4, 5, 7, 8, 9 have been deposited as individual Excel files in the ETH Zurich Research Collection (DOI: 10.3929/ethz-b-000409545).

Field-specific reporting

Please select the one below that is the best fit for your research. If you are not sure, read the appropriate sections before making your selection.

[x] Life sciences [ ] Behavioural & social sciences [ ] Ecological, evolutionary & environmental sciences

For a reference copy of the document with all sections, see nature.com/documents/nr-reporting-summary-flat.pdf

Life sciences study design

All studies must disclose on these points even when the disclosure is negative.

Sample size	The number of mice and cell cultures used for our studies was mainly based on our long-term experience with wound healing and cell culture experiments. Most of the differences that we observed were statistically significant, demonstrating the suitability of the sample size.
Data exclusions	No data were excluded from the analysis.
Replication	The results reported in this manuscript were reproducible in independent experiments as stated in the legends. No experiment is shown where data from a first experiment were not reproducible in another experiment.
Randomization	Allocation was random in all experiments.
Blinding	For the biomechanical experiments we had to determine the genotype of the mice prior to analysis, and the genotype of the activin-transgenic mice is obvious from their macrocopic appearance; however, analysis of this data was performed blinded by the investigators. All other experiments were performed blinded by the investigators during acquisition and analysis.

Reporting for specific materials, systems and methods

We require information from authors about some types of materials, experimental systems and methods used in many studies. Here, indicate whether each material, system or method listed is relevant to your study. If you are not sure if a list item applies to your research, read the appropriate section before selecting a response.

Materials & experimental systems

n/a	Involved in the study	
[ ]	[x]	Antibodies
[ ]	[x]	Eukaryotic cell lines
[x]	[ ]	Palaeontology
[ ]	[x]	Animals and other organisms
[x]	[ ]	Human research participants
[x]	[ ]	Clinical data

Methods

n/a	Involved in the study	
[x]	[ ]	ChIP-seq
[ ]	[x]	Flow cytometry
[x]	[ ]	MRI-based neuroimaging

Antibodies

Antibodies used	For FACS: CD140a-APC (#135908, clone APA5; BioLegend), CD45-Pacific blue (#103126, clone 30-F11; BioLegend), CD11b-BV711 (#101242, clone M1/70; Biolegend), F4/80-PE (#123110, clone BM8; Biolegend)<br>For ChIP: rabbit anti-Histone H3 (#ab1791; Abcam), rabbit anti-Smad 2/3 (#D7G7; Cell Signaling), normal rabbit IgG (#2729; Cell Signaling)<br>For cell culture staining: goat anti-fibronectin (#sc-6952; Santa Cruz), goat anti-collagen I (#1310-01; SouthernBiotech), rabbit anti-fibronectin (#ab2413; Abcam), rabbit anti-periostin (#ab14041; Abcam), goat anti-asporin (#ab31303; Abcam)<br>For tissue staining: rabbit anti-fibronectin (#ab2413; Abcam), rabbit anti-periostin (#ab14041; Abcam), goat anti-asporin (#ab31303; Abcam)

Validation

For FACS: all monoclonal antibodies have been validated by the manufacturer (Biolegend) and by multiple citations for mouse reactivity and for use in FACS.
For ChIP: all antibodies have been validated by the manufacturers (Abcam and Cell Signaling) and by multiple citations for mouse reactivity and for use in ChIP; normal rabbit IgG (#2729; Cell Signaling) is routinely used as a non-specific IgG control in ChIP.
For immunostaining: all antibodies used with human cells have been validated by the manufacturers (Santa Cruz and SouthernBiotech) and by multiple citations for human reactivity and for use in immunofluorescence staining; all antibodies used with mouse cells and tissues have been validated by the manufacturers (SouthernBiotech and Abcam) and by multiple citations for mouse reactivity and for use in immunofluorescence staining.
Negative controls for all antibodies were performed (e.g. Supplementary Figure 6c).

Eukaryotic cell lines

Policy information about cell lines

Cell line source(s)

Primary fibroblasts were isolated from neonatal (P2.5) or adult mouse skin or from 5-day wounds of adult mice. Passages 0-3 were used for experiments. Immortalized skin fibroblasts were initially isolated from PDGFRα-eGFP transgenic mice and spontaneously immortalized via serial passaging.
Human primary foreskin fibroblasts were kindly provided by Dr. Hans-Dietmar Beer, University Hospital Zurich. The foreskin had been collected with informed written consent of the parents and upon approval by the local ethics committee.
HaCaT cells (immortalized human keratinocytes) were directly obtained from the owner, Prof. Petra Boukamp (Boukamp, et al. J Cell Biol (1988)).
All cells were maintained in Dulbecco modified Eagle`s medium (DMEM) supplemented with 10% FBS/penicillin/streptomycin and passaged prior to confluency. Cells were starved for 24 h in DMEM/1% FBS before any treatment experiments.

Authentication

The only established cell line used in this study (HaCaT cells) was directly obtained from the owner, Prof. Petra Boukamp (Boukamp, et al. J Cell Biol (1988)), whose laboratory maintains the cell line as authentic. Immortalized mouse fibroblasts were generated in our laboratory via serial passaging of primary mouse fibroblasts, and they are continually authenticated via analysis of their fibroblastic morphology and high expression of typical fibroblast markers (e.g. vimentin and collagen).

Mycoplasma contamination

All primary cells and cell lines were tested negative for mycoplasma contamination.

Commonly misidentified lines
(See ICLAC register)

No commonly misidentified cell lines were used in the study.

Animals and other organisms

Policy information about studies involving animals; ARRIVE guidelines recommended for reporting animal research

Laboratory animals

Act mice, which express the INHBA subunit under control of the keratin 14 promoter (CD-1 genetic background) (Munz, et al. EMBO J (1999)), were mated with C57BL/6 mice, and their F1 progeny was used for FACS-sorting and RNA sequencing. For all other experiments we used mice in pure CD-1 background. Wild-type littermates were used as controls. Only female mice were used for experiments, aged 8-12 weeks. Mice were housed under specific pathogen-free (SPF) conditions under 12h/12h light/dark cycle and received food and water ad libitum. Mouse maintenance and all animal experiments had been approved by the local veterinary authorities (Kantonales Veterinäramt Zürich, Switzerland).

Wild animals

The study did not involve wild animals.

Field-collected samples

The study did not involve samples collected in the field.

Ethics oversight

All animal experiments had been approved by the local veterinary authorities (Kantonales Veterinäramt Zürich, Switzerland).

Note that full information on the approval of the study protocol must also be provided in the manuscript.

Flow Cytometry

Plots

Confirm that:

☒ The axis labels state the marker and fluorochrome used (e.g. CD4-FITC).

☒ The axis scales are clearly visible. Include numbers along axes only for bottom left plot of group (a 'group' is an analysis of identical markers).

☒ All plots are contour plots with outliers or pseudocolor plots.

☒ A numerical value for number of cells or percentage (with statistics) is provided.

Methodology

Sample preparation

Skin was excised from unwounded back skin and the underlying fat was removed. Wounds at 3-, 5- and 7-days post-injury were excised using a 4 mm biopsy punch to exclude most of the surrounding unwounded tissue. For analysis and sorting, four 5-day wounds per mouse were pooled. Wounds and unwounded skin were stored in ice-cold RPMI-1640 medium and immediately

processed. Tissue samples were minced into small pieces followed by incubation in 2 ml of medium containing 0.25 mg/ml Liberase TL (Roche), 0.25 mg/ml DNase I (Sigma) and 7.5 mM MgCl2 for 1 h at 37°C while shaking at 100 rpm. Next, the cell suspension was passed through a 70 µm cell strainer and washed with PBS containing 0.25 mg/ml DNase I and 7.5mM MgCl2. Cells were centrifuged for 10 min at 1200 rpm and resuspended in FACS buffer (0.5% BSA, 5 mM EDTA in 1X PBS) containing FcBlock (1:200; BD BioSciences, San Jose, CA) and incubated for 10 min on ice. Cells were centrifuged for 5 min at 2000 rpm and re-suspended with FACS buffer containing antibodies against different cell surface markers: CD140a-APC (#135908, clone APA5, 1:200; BioLegend, San Diego, CA), CD45-Pacific blue (#103126, clone 30-F11, 1:600; BioLegend), CD11b-BV711 (#101242, clone M1/70, 1:500; Biolegend) and F4/80-PE (#123110, clone BM8, 1:200; Biolegend). After a 30 min incubation on ice, stained cells were washed with FACS buffer. To exclude non-viable cells, Sytox Green (1:1000, Invitrogen, Carlsbad, CA) was used according to the manufacturer's instructions.

Instrument

Cells were either analyzed using the BD LSRII Fortessa or sorted/analyzed using the BD FACSAria IIIu.

Software

FACSDiva software version 6 (BD Pharmingen, San Diego, CA) for acquisition and sorting; FlowJo software version X (Tree Star Inc, Ashland, OR) for post-acquisition analysis.

Cell population abundance

Fluorescence emission compensation was performed using compensation beads (BD Biosciences). Staining and gating controls included isotype control and fluorescence minus one (FMO) samples. Compensation adjustment, gating and cell population abundance analysis during sorting was performed using FACSDiva software version 6, whereas post-acquisition compensation adjustment, gating and cell population abundance analysis was performed using FlowJo software version X.

Skin/wound fibroblasts (Figure 2): For cell analysis and sorting, fibroblasts were defined as CD140a+ CD45- CD11b- F4/80- live cells. Re-analysis of sorted cells showed >90% purity of all CD140a+ CD45- CD11b- F4/80- samples.

Keratinocyte/fibroblast co-cultures (Figure 5): For cell analysis and sorting, fibroblasts were defined as GFP+ live cells, while keratinocytes were defined as GFP- cells. Re-analysis of sorted cells showed >99% purity of all GFP+ samples (shown in Supplementary Figure 5g).

Gating strategy

Skin/wound fibroblasts (Figure 2): The gating strategy is illustrated in Figure 2a. Fibroblasts were defined as CD140a+ CD45- CD11b- F4/80- live cells.

Keratinocyte/fibroblast co-cultures (Figure 5): The gating strategy is illustrated in Supplementary Figure 5g. For cell analysis and sorting, fibroblasts were defined as GFP+ live cells, while keratinocytes were defined as GFP- cells.

☒ Tick this box to confirm that a figure exemplifying the gating strategy is provided in the Supplementary Information.
